# Comparative Effectiveness of Vonoprazan Dosing Strategies in Bismuth-Based Quadruple Therapy for *Helicobacter pylori* Eradication: A Prospective Non-inferiority Cohort Study

**DOI:** 10.5152/tjg.2026.25645

**Published:** 2026-02-13

**Authors:** Lingling Yan, Rong Lin, Yu Zhang, Shanjing Xu, Xianbin Zhou, Jiali Ma, Lina Fang

**Affiliations:** 1 Zheijang Clinovation Pride, Zhejiang, China; 2 Deparment of Gastroenterology, Taizhou Hospital of Zhejiang Province Affiliated to Wenzhou Medical University, Zhejiang, China; 3 Zhejiang Provincial Clinical Research Center for Digestive Diseases, Zhejiang, China; 4 Deparment of Internal Medicine, Linhai Hospital of Traditional Chinese Medicine, Zhejiang, China; 5 Endoscopic Center, Taizhou Hospital of Zhejiang Province Affiliated to Wenzhou Medical University, Zhejiang, China

**Keywords:** Cost-effectiveness ratio, eradication rate, *Helicobacter pylori*, quadruple therapy, vonoprazan

## Abstract

**Background/Aims:**

Vonoprazan 20 mg twice daily is effective for *Helicobacter pylori* (*H. pylori*) eradication, but studies on the efficacy of 20 mg once daily are limited. The efficacy, tolerability, and cost-effectiveness of bismuth-based quadruple therapy with vonoprazan 20 mg once daily were evaluated for *H. pylori* eradication.

**Materials and Methods:**

A prospective cohort study was conducted at TaiZhou Hospital of Zhejiang Province between January and September 2024. Participants were divided into 2 groups: low-dose vonoprazan (QD, 20 mg once daily) and standard-dose vonoprazan (BID, 20 mg twice daily). The remaining drugs (colloidal bismuth pectin, clarithromycin, and amoxicillin) were the same for both groups, with a treatment duration of 14 days. Urea breath tests were performed 6-8 weeks post-treatment.

**Results:**

In the intention-to-treat analysis, eradication rates were 89.2% (91/102) in the QD group and 88.9% (88/99) in the BID group, with no significant difference (*P* = .941). In the per-protocol (PP) analysis, eradication rates were 92.2% (83/90) in the QD group and 95.2% (79/83) in the BID group, also without a significant difference (*P* = .426). The incidence of adverse events was 8.9% in the QD group and 12.0% in the BID group (*P* = .497), respectively. The cost-effectiveness ratios were 2.59 for the QD group and 3.96 for the BID group in the PP analysis, respectively.

**Conclusion:**

Vonoprazan 20 mg once daily showed comparable efficacy to twice-daily dosing as part of a bismuth-based quadruple regimen for *H. pylori* eradication, with better cost-effectiveness, making it a promising option, especially in resource-limited settings.

**Main Points**

- Vonoprazan 20 mg once daily demonstrated non-inferior efficacy to the standard 20 mg twice-daily regimen in bismuth-based quadruple therapy for *Helicobacter pylori* eradication.
- Both regimens showed comparable safety profiles, with low rates of mild gastrointestinal adverse events.
- The once-daily vonoprazan regimen achieved better cost-effectiveness, making it a practical and economical treatment option in clinical practice, especially for resource-limited settings.

## Introduction

*Helicobacter pylori* (*H. pylori*) is a type of Gram-negative microaerophilic bacterium that can be transmitted between humans and is estimated to infect approximately 43.9% of the global population.^1^^,^^2^ *Helicobacter pylori* infection has been classified as a group 1 carcinogen by the World Health Organization and is closely associated with chronic gastritis, dyspepsia, gastric ulcer, duodenal ulcer, gastric cancer, and mucosa-associated lymphoid tissue lymphoma.^3^^,^^4^ Eradication of *H. pylori* significantly alleviates gastric mucosal inflammation, promotes ulcer healing, and reduces the risk of gastric cancer.^5^ Furthermore, this is a cost-effective strategy for gastric cancer prevention.^6^

However, *H. pylori* eradication therapies face the challenge of declining eradication rates, primarily due to antibiotic resistance and, to a lesser extent, the impact of acid-suppressive agents.^7^ To date, international guidelines and consensus conferences have recommended bismuth-based quadruple therapy (2 antibiotics, bismuth, and an acid inhibitor) as the first-line treatment for *H. pylori* infection in regions with high antibiotic resistance.^8^ Proton pump inhibitors (PPIs) are the most commonly used acid-suppressing agents in China. However, their efficacy largely depends on CYP2C19 genetic polymorphism, and rapid clearance of PPIs in fast-metabolizing individuals may reduce the eradication rate of *H. pylori*.^9^

Vonoprazan is a novel potassium-competitive acid blocker (P-CAB) that exerts its acid-suppressing effect by competitively and reversibly inhibiting H+/K+-ATPase activity, and was first approved in Japan in 2015.^10^^,11^ Compared with PPIs, vonoprazan provides more rapid and sustained acid inhibition and is not affected by CYP2C19 polymorphism.^12^ Previous studies have reported that first-line therapies based on vonoprazan (20 mg twice daily) achieved higher *H. pylori* eradication rates than those based on PPIs.^13^^,^^14^ In addition, previous studies have shown that vonoprazan 20 mg once daily exhibits efficacy comparable to esomeprazole 20 mg twice daily and is not influenced by CYP2C19 polymorphism.^15^ However, few studies have investigated the effectiveness of vonoprazan 20 mg once daily as a critical element of *H. pylori* therapy.

Therefore, it was hypothesized that vonoprazan 20 mg once daily might serve as an effective part of *H. pylori* eradication therapy. To evaluate this hypothesis, a prospective cohort study was conducted comparing the efficacy and safety of 2 first-line quadruple regimens: vonoprazan 20 mg once daily and vonoprazan 20 mg twice daily.

## Materials and Methods

### Participants

This was a single-center, prospective cohort study. A total of 201 patients diagnosed with *H. pylori* infection and treated at TaiZhou Hospital of Zhejiang Province between January 1, 2024, and September 30, 2024, were included. According to the vonoprazan regimen selected by the attending physician, patients were divided into 2 groups: the once-daily 20 mg regimen (QD group) and the twice-daily 20 mg regimen (BID group). Ethical committee approval was received from the Ethics Committee of TaiZhou Hospital of Zhejiang Province (Approval no: K20250231, Date: February 28, 2025). All patients in this study signed written informed consent forms.

The inclusion criteria were as follows: 1) no prior *H. pylori* eradication therapy; 2) age between 18 and 75 years old; 3) *H. pylori* infection confirmed by a ^13^C or ^14^C urea breath test (UBT) or a histopathological examination; 4) treatment regimen consisting of P-CAB, bismuth pectin, amoxicillin, and clarithromycin; and 5) willingness to attend the follow-up ^14^C-UBT or the ^13^C-UBT after treatment completion. Patients were excluded based on the following criteria: 1) allergy to P-CAB, bismuth pectin, amoxicillin, or clarithromycin; 2) severe hepatic or renal dysfunction; 3) pregnancy or lactation; 4) a history of gastric malignancy; and 5) unwillingness to comply with the follow-up assessments.

### Therapeutic Regimens

Both groups received 14-day bismuth-based quadruple therapy. The medications used included vonoprazan (Takeda Pharmaceutical Co., Ltd., Tianjin, China; 20 mg/tablet), amoxicillin capsules (Aomei Pharmaceutical Co., Ltd., Hong Kong, China; 250 mg/capsule), colloidal bismuth pectin capsules (Shanxi Ante Biopharmaceutical Co., Ltd., Shanxi, China; 100 mg/capsule), and clarithromycin tablets (Zhejiang Beid Pharmaceutical Co., Ltd., Zhejiang, China; 250 mg/tablet).

The QD group received vonoprazan (20 mg once daily), colloidal bismuth pectin capsules (200 mg twice daily), amoxicillin capsules (1000 mg twice daily), and clarithromycin tablets (500 mg twice daily); while the BID group received vonoprazan (20 mg twice daily), colloidal bismuth pectin capsules (200 mg twice daily), amoxicillin capsules (1000 mg twice daily), and clarithromycin tablets (500 mg twice daily). Vonoprazan should be administered regularly, regardless of the meal, while colloidal bismuth pectin should be administered 30 minutes before meals, and amoxicillin and clarithromycin should be administered after meals. Medication guidance and medication cards (for recording adherence and adverse drug reactions) were provided at baseline.

### Study Outcomes

The primary outcome of this study was the rate of *H. pylori* eradication in both groups. At 6-8 weeks after completing the eradication regimen, patients underwent a ^13^C-UBT or ^14^C-UBT. A negative result was considered evidence of successful eradication. The test was considered positive when the ^13^C-UBT (delta over baseline, DOB) was >5‰ or the ^14^C-UBT was >100 disintegrations per minute; otherwise, it was considered negative. Participants were required to refrain from using PPIs or P-CAB for at least 2 weeks before the breath test and from taking any antibiotics for at least 4 weeks prior.

Secondary outcomes included adverse events (AEs) and treatment adherence. The AEs occurring during treatment, such as abdominal pain, bloating, diarrhea, nausea, vomiting, constipation, rash, dizziness, and loss of appetite, were documented through telephone follow-up after treatment completion. These events were classified as mild (not affecting the normal function), moderate (partially affecting the normal function), and severe (significantly impairing the normal function). Adherence was considered good if the medication was taken for the full 14-day course, without any missed or incorrect doses.

### Cost-Effectiveness Analyses

To compare the pharmacoeconomic differences between the treatment regimens, this study conducted a cost-effectiveness analysis. However, only the direct medical costs associated with the treatment drugs (acid suppressants, antibiotics, and bismuth agents) were evaluated. Treatment efficacy was measured based on the *H. pylori* eradication rate. The cost-effectiveness ratio (CER) was calculated as the direct medical cost divided by the *H. pylori* eradication rate.

### Sample Size Calculation

A previous study reported a 94.9% eradication rate with similar first-line *H. pylori* treatment regimens.^16^ Based on this, the same eradication rate was assumed for both groups and adopted a non-inferiority design. A non-inferiority margin of 10% was established with 80% power and a one-sided alpha of 0.025. These parameters indicated a minimum requirement for 152 patients (76 per group). Considering a 15% dropout rate, the final sample size was adjusted to 178. Sample size calculations were conducted using the PASS 2021 software program (Kaysville, UT, USA).

### Statistical Analysis

Continuous variables with a normal distribution are presented as mean ± standard deviation (x̅ ± s) and were compared between groups using an independent sample *t*-test. Skewed quantitative data are presented as medians with interquartile ranges (Q1, Q3), and comparisons were made using the Mann–Whitney *U*-test. Categorical data are reported as frequencies (%), and group differences were assessed using the chi-square test or Fisher’s exact test. Statistical significance was defined as *P* < .05.

The intention-to-treat (ITT) analysis encompassed all enrolled patients, with those failing to undergo the UBT at 6-8 weeks post-therapy classified as eradication failures. In contrast, the per-protocol (PP) analysis excluded individuals who discontinued treatment due to AEs or were lost to follow-up.

All statistical analyses were performed using the SPSS software program version 26.0 (IBM SPSS Corp.; Armonk, NY, USA).

## Results

### Baseline Characteristics of the Patients

A total of 201 patients were enrolled at TaiZhou Hospital between January and September 2024. Among them, 12 patients in the QD group experienced missed or incorrect medication administration, accounting for 11.8% (12/102), whereas 16 patients in the BID group experienced the same issue, accounting for 16.2% (16/99).

There were no significant differences observed between the QD and BID groups regarding age, sex distribution, body mass index, body surface area, smoking, alcohol consumption, or educational level distribution (all *P* > .05) (Table 1).

### *Helicobacter pylori* Eradication Rates

In the ITT analysis, *H. pylori* eradication rates were 89.2% (91/102) in the QD group and 88.9% (88/99) in the BID group, with no statistically significant differences between the groups (*P* = .941). By PP analysis, *H. pylori* eradication rates were 92.2% (83/90) in the QD group and 95.2% (79/83) in the BID group, with no statistically significant differences (P = 0.426) (Table 2).

### Adverse Events

In the ITT population, AEs were reported in 10 patients in the QD group, with an incidence of 9.8% (10/102), including 5 patients who experienced ≥2 AEs. In the BID group, AEs occurred in 12 patients, with an incidence of 12.1% (12/99), including 4 patients who experienced ≥2 AEs. The difference in AE incidence between the 2 groups was not statistically significant (*P* = .599). The incidence of mild AEs in the QD and BID groups was 8.8% (9/102) and 10.1% (10/99), respectively, whereas the incidence of moderate AEs was 1.0% (1/102) and 2.0% (2/99), respectively (Table 2).

Among the PP population, AEs were reported in 8 patients in the QD group, with an incidence of 8.9% (8/90), including 3 patients who experienced ≥2 AEs. In the BID group, AEs occurred in 10 patients, with an incidence of 12.0% (10/83), including 3 patients who experienced ≥2 AEs. The difference in AE incidence between the 2 groups was also not statistically significant (*P* = .497). The incidence of mild AEs in the QD and BID groups was 7.8% (7/90) and 10.8% (9/83), respectively, whereas the incidence of moderate AEs was 1.1% (1/90) and 1.2% (1/83), respectively (Table 2).

The most commonly reported AEs included abdominal pain, bloating, diarrhea, nausea, vomiting, constipation, dizziness, anorexia, and rash (Table 2). Among these, 1 patient in the BID group developed an allergic reaction on the eighth day of treatment, which resolved following self-discontinuation of the medication and administration of antiallergic drugs. All other AEs resolved spontaneously within 1 week after treatment completion.

### Cost-effectiveness Analyses

In the ITT analysis, the medication costs for the QD and BID treatment groups were 238.62 RMB/patient (32.8 USD) and 377.22 RMB/patient (51.9 USD), respectively, with CERs of 2.68 and 4.24. In the PP analysis, the CERs for the QD and BID groups were 2.59 and 3.96, respectively (Table 3). The QD group exhibited a more favorable CER than the BID group did.

## Discussion

Currently, PPI-based treatment regimens face the challenge of declining *H. pylori* eradication rates, primarily because of the widespread presence of antibiotic resistance and insufficient acid suppression.^17^ Studies have shown that many PPI-based regimens fail to achieve the desired 90% eradication rate.^18^ The slow onset of action and short half-life of PPIs lead to insufficient and transient acid suppression, and the efficacy of acid suppression is influenced by CYP2C19 genetic polymorphisms, which may weaken the overall effectiveness of *H. pylori* eradication therapy.^19^ To address this issue, previous studies have proposed various strategies to improve *H. pylori* eradication, including high-dose antibiotic and high-dose PPI regimens.^20^ Potent acid suppressants may enhance eradication regimens, as *H. pylori* can resume replication when gastric pH exceeds 6, making the bacterium more vulnerable to antimicrobial treatment.^21^ Vonoprazan, a novel acid suppressant, offers advantages over PPIs, such as a rapid onset, long-lasting effects, and independence from CYP2C19 polymorphisms.^15^ A meta-analysis of 101 randomized controlled trials involving 21 745 patients demonstrated that bismuth-based quadruple therapy with vonoprazan (20 mg twice daily) achieved higher eradication rates compared to bismuth-based quadruple therapy or high-dose amoxicillin dual therapy with PPIs.^20^ However, research on the effectiveness of bismuth-based quadruple therapy with a lower dose of vonoprazan (20 mg once daily) for *H. pylori* eradication is limited.

Lu et al^16^ performed a non-inferiority, randomized controlled trial to assess the effectiveness of vonoprazan 20 mg once daily in bismuth-based quadruple therapy compared to a PPI-based bismuth quadruple regimen. The results showed that vonoprazan quadruple therapy was non-inferior to the esomeprazole-based quadruple regimen in terms of efficacy. Furthermore, several studies have demonstrated that vonoprazan 20 mg twice daily, in dual, triple, or quadruple regimens, achieves superior *H. pylori* eradication rates compared with PPI-based therapies.^13^^,^^14^^,^^20^^,^^22^ However, to date, no studies have directly compared the effectiveness of vonoprazan 20 mg once daily with vonoprazan 20 mg twice daily in a bismuth-based quadruple regimen.

The findings of this study showed that vonoprazan 20 mg QD is non-inferior to vonoprazan 20 mg BID in the eradication of *H. pylori*, as evidenced by comparable eradication rates between the 2 regimens. In addition, both regimens demonstrated favorable safety profiles, with no significant differences in AE rates. Furthermore, the cost-effectiveness analysis suggests that the QD regimen may offer a more economical approach to *H. pylori* eradication than the BID regimen.

In the present study, no statistically significant difference in *H. pylori* eradication rates was observed, with the low-dose vonoprazan regimen achieving a rate of 92.2% and the standard-dose regimen achieving a rate of 95.2% in the PP population. Several possible reasons for this result are proposed. First, consensus guidelines suggest that the optimal eradication of *H. pylori* infection requires achieving a gastric pH between 6 and 7, a range in which *H. pylori *is in its proliferative phase and particularly susceptible to antibiotics.^5^ The median intragastric pH for vonoprazan 20 mg twice daily was 6.8, while for 20 mg once daily it was 6.5.^15^ Therefore, it is believed that a low-dose vonoprazan regimen is sufficient to meet the acid suppression requirements for *H. pylori* eradication. Second, this study was a single-center investigation with a small sample size, which may introduce potential bias; thus, further validation through multicenter, large-scale, prospective, randomized controlled trials is needed. The results of this study are consistent with a previous study on the use of a low-dose vonoprazan-based quadruple regimen for *H. pylori* eradication, although the eradication rate in the low-dose group in the study (92.2%) was lower than that reported in an earlier study (97.4%).^16^ This discrepancy may be attributed to the use of furazolidone instead of clarithromycin as an antibiotic in the quadruple regimen in a previous study. Although furazolidone is associated with lower resistance rates and costs than clarithromycin, it also has the potential for serious side effects.^23^ Therefore, this study employed a traditional antibiotic regimen that combined amoxicillin and clarithromycin, which resulted in an acceptable *H. pylori* eradication rate.

Interestingly, although there was no significant difference in eradication rates between the low- and standard-dose treatment groups, a notable increasing trend in eradication rates was observed in the standard-dose group among patients in the PP population (88.9% vs. 95.2%). This suggests that, while the low-dose regimen is effective, patients who strictly adhere to the treatment may experience a slight additional benefit from the standard-dose regimen. However, this difference was not statistically significant, indicating that, for most patients, the once-daily dosing regimen was sufficient to achieve optimal eradication outcomes.

In terms of safety, the incidence of AEs was similar between the 2 groups in the present study, with no significant differences observed in either the ITT population or in the PP population. The most commonly reported AEs were mild gastrointestinal symptoms, such as abdominal pain, bloating, and diarrhea, which are typically associated with changes in the gut microbiota due to antibiotic use.^24^ Notably, the incidence of moderate or severe AEs was low, indicating that the patients generally tolerated both treatment regimens well. These findings align with the established safety profile of vonoprazan, as previous studies have demonstrated that vonoprazan is well tolerated and has a favorable safety profile compared with traditional PPIs.^25^ Another important aspect of this study was the cost-effectiveness analysis, which revealed that the low-dose treatment regimen was more cost-effective than the standard-dose regimen. This finding is particularly relevant for healthcare resource allocation, as it suggests that patients can achieve similar clinical outcomes with a more economical treatment option. The improved cost-effectiveness of the low-dose regimen supports its potential as the preferred treatment choice in clinical practice, particularly in resource-limited settings.

One limitation of the study was its non-randomized controlled design, which may lead to selection bias and confounding factors that could influence the results. Furthermore, the study was conducted at a single center, which may restrict the generalizability of the findings to a broader population. To validate these results and further assess the comparative efficacy, safety, and cost-effectiveness of a 20 mg/day-based regimen for *H. pylori* eradication, future prospective multicenter randomized controlled trials are necessary.

In conclusion, the efficacy of vonoprazan 20 mg once daily is non-inferior to that of 20 mg vonoprazan twice daily in a bismuth-based quadruple regimen. The low-dose regimen exhibited favorable tolerability and safety profiles. In addition, a low-dose regimen offers improved cost-effectiveness, making it a promising option for *H. pylori* treatment, particularly in resource-limited settings. Further multicenter, large-scale, prospective, randomized controlled trials are needed to validate these findings.

## Figures and Tables

**Table 1.** Comparison of Clinical Characteristics and Treatment Outcomes Between QD and BID Groups

**Variables**	**QD Group ** **N = 102**	**BID Group ** **N = 99**	***P ***
Age, years	51.50 (43.00-59.00)	53.00 (42.00-63.00)	.407
Sex Female Male	45 (44.1) 57 (55.9)	48 (48.5) 51 (51.5)	.535
BMI, kg/m^2^	23.74 (22.31-25.39)	23.44 (22.03-25.65)	.540
Body surface area, m^2^	1.70 ± 0.16	1.71 ± 0.15	.768
Cigarette smoking Yes No	18 (17.6) 84 (82.4)	20 (20.2) 79 (79.8)	.644
Alcohol consumption Yes No	13 (12.7) 89 (87.3)	22 (22.2) 77 (77.8)	.077
Education level Illiterate Primary school Middle school High school or above	7 (6.9) 24 (23.5) 49 (48.0) 22 (21.6)	10 (10.1) 35 (35.4) 37 (37.4) 17 (17.1)	.183
Missed or incorrect medication administration Yes No	12 (11.8) 90 (88.2)	16 (16.2) 83 (83.8)	.288

Quantitative data expressed as medians with interquartile range or mean ± standard deviation. Qualitative data expressed as the frequency (%).

BID group, twice-daily group; QD group, once-daily group.

**Table 2.** Primary and Secondary Outcomes

	**Intention-to-treat Analysis**			**Per-protocol Analysis**		
	**QD Group ** **N = 102**	**BID Group ** **N = 99**	***P***	**QD Group** **N = 90**	**BID Group** **N = 83**	***P***
Successful eradication Yes No	91 (89.2) 11 (10.8)	88 (88.9) 11 (11.1)	.941	83 (92.2) 7 (7.8)	79 (95.2) 4 (4.8)	.426
Adverse events Yes No	10 (9.8) 92 (90.2)	12 (12.0) 87 (87.9)	.599	8 (8.9) 82 (91.1)	10 (12.0) 73 (88.0)	.497
Adverse events Abdominal pain Bloating Diarrhea Nausea Vomiting Constipation Dizzines Anorexia Rash	2 2 3 4 2 0 2 1 0	4 2 2 0 1 3 1 2 1		1 0 3 3 2 0 2 1 0	4 1 2 0 1 3 1 1 0	

Qualitative data expressed as the frequency (%).

BID group, twice-daily group; QD group, once-daily group.

**Table 3.** Cost-effectiveness Ratios

	**Group**	**Total Cost**	**Eradication Rates**	**CER**
Intention-to-treat analysis	QD group BID group	238.62 377.22	89.2% (91/102) 88.9% (88/99)	2.68 4.24
Per-protocol analysis	QD group BID group	238.62 377.22	92.2% (83/90) 95.2% (79/83)	2.59 3.96

BID group, twice-daily group; CER, Cost-effectiveness ratio; QD group, once-daily group.

## Data Availability

The data that support the findings of this study are available on request from the corresponding author.
